# Adaptation to San Martín Quechua and psychometric analysis of the Patient Health Questionnaire (PHQ-9) in Peruvian adults

**DOI:** 10.1186/s40359-026-04653-9

**Published:** 2026-05-02

**Authors:** Julio Cjuno, Heber Gomez-Hurtado, Cristian Shupingahua-Jimenez, Jessica Aranda-Turpo, Félix Julca Guerrero, Nilo Velásquez-Castillo, Eddy Wildmar Aquize Anco, Janina Bazalar-Palacios, Juan Carlos Bazo-Alvarez

**Affiliations:** 1 Universidad Peruana Unión, Unidad de Posgrado de Psicología, Lima, Perú; 2 Universidad Tecnológica del Perú, Lima, Perú; 3 Universidad Peruana Unión, Escuela Profesional de Psicología, Tarapoto, Perú; 4 Universidad Nacional Santiago Antúnez de Mayolo, Huaraz, Perú; 5 Universidad Católica Los Ángeles de Chimbote, Chimbote, Perú; 6 Universidad Científica del Sur, Lima, Perú; 7 Escuela de Psicología, Universidad Cesar Vallejo, Trujillo, Peru; 8 Research Department of Primary Care and Population Health, University College London (UCL), London, UK

**Keywords:** Depression, Patient Health Questionnaire, Indigenous peoples, Quechua people (Source: Mesh)

## Abstract

**Objective:**

To culturally adapt the Patient Health Questionnaire-9 (PHQ-9) for San Martín Quechua and to evaluate its structural validity, reliability, and measurement invariance in Peruvian adults.

**Methods:**

A psychometric study was conducted on a non-probabilistic sample of 309 Quechua-speaking adults from the San Martín region of Peru, including both men and women aged 18 to 59 years. The study was carried out in two phases. The first phase involved the translation and cultural adaptation of the original English PHQ-9 into the Quechua variant spoken in San Martín. The second phase tested how well the adapted PHQ-9 worked by using different methods: confirmatory factor analysis (CFA) to examine its internal structure, multi-group CFA to assess measurement invariance across groups, the Average Variance Extracted (AVE) to evaluate construct representation, and reliability estimation. Convergent validity was also examined through correlations with related measures.

**Results:**

A unidimensional model of the PHQ-9 Quechua San Martín showed adequate fit (CFI = 0.996, TLI = 0.994, SRMR = 0.048, RMSEA = 0.051) and high reliability (α = 0.86; Ω = 0.87). Measurement invariance was supported across sociodemographic groups (ΔCFI and/or ΔRMSEA < 0.01). Convergent validity was supported by an adequate AVE (0.53). Additionally, PHQ-9 scores correlated moderately with the Generalized Anxiety Disorder-7 and weakly and negatively with the Well-Being Index, supporting convergent validity based on relations with other variables.

**Conclusions:**

The San Martín Quechua PHQ-9 is a valid, reliable, and at least partially invariant single-factor instrument for assessing depressive symptoms in Quechua-speaking adults. It may be useful as a screening tool in similar populations.

## Introduction

Depression is a mental health disorder that affects more than 280 million people worldwide [34]. This condition significantly impairs individuals’ quality of life and represents a major burden for healthcare systems due to its high comorbidity with other physical and mental illnesses [24]. In Peru, it is estimated that approximately one in every 20 people experiences severe depressive symptoms [42, 44], with even higher prevalence in regions marked by poverty [25], such as rural communities and culturally diverse groups—particularly speakers of Indigenous languages [11]. The Quechua-speaking population is especially notable for high rates of depressive mood, linked to factors such as unequal access to healthcare, low socioeconomic status, high alcohol consumption, racial discrimination, past adverse experiences, and language barriers in medical settings [38]. This situation emphasizes the need to implement culturally safe and linguistically appropriate strategies within the Peruvian healthcare system [7].

Quechua is a broad family of languages composed of 13 or more modern varieties [36], grouped into three main subfamilies: Central, Southern, and Northern Quechua [40]. In Peru, there are three subgroups of Northern Quechua, the San Martín variety, also known as Lamisto Quechua, spoken primarily in the provinces of Lamas (Wayku and nearby settlements), San Martín (Chazuta district along the Huallaga River), and El Dorado (San José de Sisa district) [48]. This language variant has unique sound features like voiced stops and phoneme fusion, as well as specific ways to show possession and plural forms [1, 8]. These cultural and linguistic particularities point to the importance of appropriately adapting assessment tools for depression in this community. Cultural worldviews also shape how depressive symptoms are understood and expressed in Indigenous populations. Emotional distress may be conveyed through culturally specific idioms and relational frameworks that differ from Western psychiatric models [13]. Therefore, cross-cultural adaptation requires ensuring conceptual equivalence, as cultural meanings can influence how individuals interpret and respond to questionnaire items, potentially affecting the validity of the instrument [15].

The Patient Health Questionnaire-9 (PHQ-9) is the most widely used depression screening instrument globally. At the moment, the PHQ-9 has been translated and validated in over 20 languages [14], including lesser-used languages like Swahili [31], Māori [3], and various Indigenous Latin American languages such as Bolivian Quechua [5] and three Peruvian varieties: Central Quechua, Chanca, and Cuzco-Collao [12]. Its brevity and alignment with diagnostic criteria for major depression, make it well-suited for both clinical and community settings. Adapting the PHQ-9 to the Quechua of San Martín is a key step aligned with the World Health Organization’s initiative to promote mental health equity among Indigenous populations [33].

The choice of a unidimensional model for the PHQ-9 is supported by its original development [39]. In addition, previous cultural adaptation studies in Indigenous languages, such as Bolivian Quechua [5], as well as adaptations to three Peruvian Quechua varieties (Chanca, Cuzco-Collao, and Central Quechua) [12], and the Peruvian Spanish version validated in a large population-based sample (*n* = 30,449) [43], have consistently reported adequate internal structure validity, reliability, and measurement invariance. Furthermore, empirical evidence indicates that two-factor solutions are not consistently replicated and often exhibit high correlations between factors, which calls into question their theoretical distinctiveness. Moreover, in cross-cultural validation studies, bidimensional models have not demonstrated clear advantages in terms of model fit or interpretability over the unidimensional solution [14]. Based on these premises, it is hypothesized that the PHQ-9 will retain a unidimensional structure in its adaptation to San Martín Quechua.

In response, this study aimed to culturally adapt the Patient Health Questionnaire-PHQ-9 to the Quechua cultural context of a specific Peruvian population (San Martin region) and analyze the internal structure validity, reliability, and measurement invariance by sociodemographic variables.

## Methods

### Study design and setting

An instrumental study was developed in which we culturally adapted and validated the San Martin Quechua version of the PHQ-9 from English and reviewed its psychometric properties. The subject matter was an instrumental or psychometric study conducted in the department of San Martín, specifically in the province of Lamas. In this region, the Quechua-speaking community comprises approximately 82,141 individuals, primarily engaged in agriculture, domestic work, fishing, and agricultural trade [4].

### Translation and cultural adaptation

The translation process was carried out in two stages. The first stage involved forward translation, conducted independently by two native speakers of San Martín Quechua with advanced proficiency in American English. Each translator rendered the PHQ-9 from English to Quechua independently. A consensus meeting was later held between the translators and researchers to resolve discrepancies and agree on a unified version.

The second stage involved back-translation, performed independently by two additional translators who were fluent in San Martín Quechua and certified in advanced American English. They translated the instrument from Quechua back into English. Researchers and translators then reviewed the conceptual, linguistic, and idiomatic equivalences. The first Quechua version of the PHQ-9 emerged from this process and served as the basis for the subsequent step.

Using the Delphi method [32], three expert judges were selected (i.e., two Quechua-speaking psychologists of this dialect and one nurse), each with at least two years of experience working with patients experiencing depressive symptoms in Quechua-speaking communities in Lamas. Each expert received an evaluation form via WhatsApp, which included the Quechua-translated version of the PHQ-9 and questions about culturally sensitive terms such as “depressed” and “hopeless” in item 2, replacing “reading a newspaper” with “listening to the radio,” and modifying the Likert scale. The interaction involved three rounds, during which researchers presented revised versions of the Quechua PHQ-9. In the third round, the experts approved the revised version and quantitatively rated the items based on relevance, representativeness, and clarity.

A virtual focus group was then conducted via Zoom, moderated by a psychologist experienced in qualitative research. Ten bilingual adults from Lamas (five men and five women, aged 18 or older, fluent in both Quechua and Spanish) participated after providing informed consent. Initially, they completed the Quechua PHQ-9 using a Google Forms link. The moderator then initiated a discussion, asking participants whether any items or terms were unclear. This led to a fluent, guided conversation. The final version of the instrument is available at 10.5281/zenodo.18072284.

### Participants and sampling

A total of 309 Quechua speakers from the San Martín variety participated in this non-probabilistic sample. The sample size exceeded the minimum required, as estimated using an online calculator for Structural Equation Modeling [2]. The calculation assumed a Comparative Fit Index (CFI) > 0.95, a statistical power of 85%, and a 10% refusal rate, yielding a minimum sample of 282 participants. The inclusion criteria specified that participants must be adults over 18 years old, of both sexes, and Quechua speakers from the province of Lamas in San Martín, Peru, who provided informed consent voluntarily. Exclusion criteria included physical or mental conditions that interfered with coherent responses or incomplete surveys.

### Instruments

Researchers used the English version of the Patient Health Questionnaire (PHQ-9), originally developed in the United States [39]. The PHQ-9 is a self-report instrument based on a unidimensional structure aligned with DSM-IV diagnostic criteria. It assesses depressive symptoms through nine items, with responses rated on a Likert scale from 0 (“not at all”) to 3 (“nearly every day”), referring to the past two weeks. Total scores range from 0 to 27, with a cut-off score of ≥ 10 indicating probable depression.

For convergent validity, the General Anxiety Disorder-7 (GAD-7) was used, which has been validated for the Peruvian adult population [42, 44]. This instrument has a unidimensional factor structure consisting of seven items referring to symptoms experienced during the past two weeks, with Likert-type response options (not at all = 0, several days = 1, more than half the days = 2, nearly every day = 3). Regarding validity evidence, it showed good model fit (CFI = 0.994; TLI = 0.991; RMSEA = 0.068; SRMR = 1.567), adequate internal consistency (ω = 0.90, α = 0.93), and a strong positive association with the PHQ-9 (*r* = 0.77).

On the other hand, the WHO-5 Well-Being Index was used, which has been adapted and validated for the Peruvian context [17]. This instrument demonstrated a unidimensional factor structure consisting of five items with Likert-type response options (0 = never, 1 = sometimes, 2 = often, 3 = always). Regarding structural validity, the measure showed good model fit (CFI = 0.998, TLI = 0.995, SRMR = 0.03, and RMSEA = 0.06) and optimal internal consistency (α = 0.84; ω = 0.84).

Sociodemographic variables were also collected, such as age (18 to 30, 31 to 59), sex (female, male), level of education (secondary or less, higher), marital status (Single/divorced/widowed, Married/cohabiting), residence (rural area, urban area).

### Procedures

Following authorization from local community leaders (e.g., heads of rural patrol groups) in Lamas, the survey was administered in paper format. A trained Quechua-speaking psychology graduate conducted face-to-face interviews during house-to-house visits. Each potential participant was informed about the study’s purpose and signed informed consent. Only those who voluntarily agreed completed the questionnaire. Data collection took place over two months, from July to August 2024.

### Statistical analyses

Descriptive statistics included absolute and relative frequencies for categorical variables. The validity of the internal structure was checked using Confirmatory Factor Analysis (CFA) with the Weighted Least Square Mean and Variance Adjusted (WLSMV) method, which is suitable for ordinal data [6]. The model’s fit was checked using several criteria: chi-square (χ^2^/df should be less than 3), Comparative Fit Index (CFI should be greater than 0.95), Tucker-Lewis Index (TLI should be greater than 0.95), Root Mean Square Error of Approximation (RMSEA should be less than 0.08), and Standardized Root Mean Square Residual (SRMR should be less than 0.08) [10].

Measurement invariance was tested using multi-group CFA, with groups based on age, sex, marital status, educational level, and residence. Differences in CFI (ΔCFI) and RMSEA (ΔRMSEA) were used to compare increasingly constrained models (configural, metric, scalar, and strict). A ΔCFI or ΔRMSEA < 0.01 indicated adequate invariance at each level [46].

Convergent validity was examined using the Average Variance Extracted (AVE) and the mean of the standardized factor loadings obtained using the CFA model with WLSMV estimation. Reliability was evaluated using Cronbach’s alpha and McDonald’s omega, with coefficients > 0.70 considered acceptable [41]. In addition, correlations among the PHQ-9, GAD-7, and WHO-5 total scores were analyzed using Spearman’s coefficient to further assess convergent validity. All statistical analyses were conducted in RStudio version 2024.09.1 using the SemTools [26], SemPlot [18], and Lavaan [37] packages.

### Ethical considerations

This study was approved by the Ethics Committee of Universidad César Vallejo (code 440-CEI-EPM-UCV-2024, from May 11, 2024). The study adhered to the principles of the Declaration of Helsinki, including informed consent, confidentiality, and equity.

## Results

Following the expert review and the focus group discussions, the consultations regarding Item 2 led to the adaptation of the term *“deprimido”* to *“llakishka,”* as this word denotes a state of profound sadness and is commonly used in these communities to express experiences associated with depression [11]. Likewise, *“sin esperanza”* was translated as *“mana kananpa,”* which refers to a person who does not expect anything good to occur in their future. In addition, the phrase *“ver televisión”* from Item 7 was replaced with *“escuchar radio,”* given that listening to radio programs in Quechua is a common daily activity among speakers of this variety. Finally, the adapted Likert scale was defined as follows: *Never* = *0, Several days* = *1, Many days* = *2, Nearly every day* = *3.*

A sample of 309 Quechua-speaking adults was obtained. Among them, 189 (61.2%) were between 18 and 29 years old, 193 (62.5%) were women, 184 (59.5%) reported having completed primary or secondary education, 171 (55.3%) identified as single, divorced, or widowed, and 165 (53.4%) resided in urban areas (Table 1).

**Table 1:** Characteristics of population from San Martin (*n* = 309)

Variables	**N**	**%**
Age		
18 to 29 years	181	58.6
30 to 59 years	128	41.4
Sex		
Female	193	62.5
Male	116	37.5
Education level		
Secondary or less	184	59.5
Higher education	125	40.5
Marital status		
Single, divorced, and widowed	171	55.3
Married and cohabiting	138	44.7
Residence		
Urban area	165	53.4
Rural area	144	46.6

### Internal structure validity

**Fig. 1:**
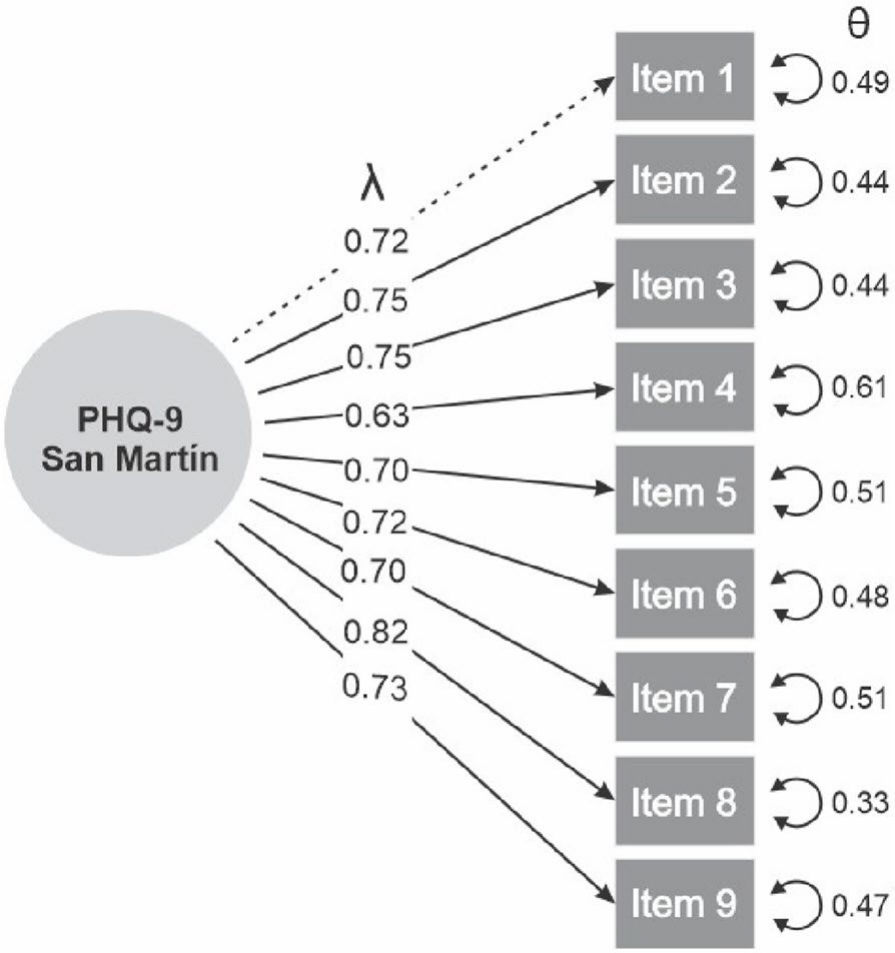
Chart of the SEM Structural Model of the PHQ-9 Quechua San Martín

The fit indices show excellent performance of the one-dimensional model of the San Martín Quechua PHQ-9, with CFI and TLI close to 1.0 and a very low SRMR. The RMSEA value, along with its 90% confidence interval (0.027–0.074), indicates a good to adequate fit (Table 2). Additionally, the single latent factor showed factor loadings ranging from a minimum of λ = 0.63 to a maximum of λ = 0.82 across the items of the San Martín Quechua version of the PHQ-9 (Fig. 1).

**Table 2:** Goodness-of-fit index of the PHQ-9 Quechua of San Martín

Model	Goodness-of-fit index	PHQ-9 Quechua (*n* = 309)
1-Dimension	X^2^ (27)	48.560
	CFI	0.996
	TLI	0.994
	SRMR	0.048
	RMSEA	0.051
	RMSEA IC 90%	0.027—0.074

*Abbreviations*: X^2^ (df) for model versus baseline, *CFI* Comparative fit index, *TLI* Tucker-Lewis’s index, *SRMR* Standardized root mean squared residual, *RMSEA* Root mean squared error of approximation

### Measurement invariance

In the multigroup CFA, metric invariance was generally supported, as indicated by minimal changes in model fit (ΔCFI ranging from − 0.003 to − 0.001). However, for age groups, the increase in RMSEA slightly exceeded the recommended threshold (ΔRMSEA = 0.016), suggesting a marginal deterioration in absolute model fit. Scalar invariance was also achieved across all groups (ΔCFI ≤ 0.007), with consistently high CFI values (0.997–0.999) and RMSEA values within acceptable ranges; in some cases, decreases in RMSEA were observed, indicating improved model fit under more constrained models. While strict invariance models showed additional increases in chi-square (up to 131.692, df = 80) and slight decreases in CFI (ΔCFI ranging from − 0.005 to − 0.007), overall model fit remained within acceptable limits (CFI ≥ 0.990; RMSEA ≤ 0.065). Taken together, these findings support the presence of at least partial measurement invariance, with particularly strong evidence for scalar invariance, allowing for meaningful comparisons of latent means across groups (Table 3).

**Table 3:** Multigroup CFA goodness-of-fit index for the San Martín Quechua PHQ-9

Group	Model	X^2^	df	CFI	ΔCFI	RMSEA	ΔRMSEA	Decision
Age	Configural	80.256	54	0.995		0.047		Accept
	Metric	105.67	62	0.992	−.003	0.063	0.016	Marginal
	Scalar	88.334	71	0.997	0.005	0.051	−.012	Accept
	Strict	125.742	80	0.992	−.005	0.051	0.000	Accept
Sex	Configural	70.071	54	0.997		0.044		Accept
	Metric	95.133	62	0.994	−.003	0.058	0.014	Accept
	Scalar	82.44	71	0.998	0.004	0.025	−.033	Accept
	Strict	103.836	80	0.996	−.002	0.025	0.000	Accept
Education	Configural	78.452	42	0.995		0.054		Accept
	Metric	95.15	62	0.994	−.001	0.059	0.005	Accept
	Scalar	87.552	71	0.997	0.003	0.040	−.019	Accept
	Strict	131.692	80	0.990	−.007	0.040	0.000	Accept
Marital status	Configural	76.403	54	0.996		0.051		Accept
	Metric	100.201	62	0.993	−.003	0.062	0.011	Accept
	Scalar	87.628	71	0.997	0.004	0.053	−.009	Accept
	Strict	121.133	80	0.992	−.005	0.053	0.000	Accept
Residence	Configural	69.891	54	0.997		0.043		Accept
	Metric	81.404	62	0.996	−.001	0.044	0.001	Accept
	Scalar	76.303	71	0.999	0.003	0.031	−.012	Accept
	Strict	87.375	80	0.999	0.000	0.031	0.000	Accept

ΔCFI ≤.010 and ΔRMSEA ≤.015 were used as criteria for invariance [9]. Negative ΔCFI values indicate a decrease in model fit. Decisions are based on joint evaluation of ΔCFI and ΔRMSEA

*RMSEA* Root Mean Square Error of Approximation, *CFI* Comparative Fit Index

### Convergent validity and reliability

Additionally, to assess convergent validity based on relationships with other variables, a moderate, positive correlation was found between the Quechua San Martín Version of the PHQ-9 and the Generalized Anxiety Disorder-7 (GAD-7) (*ρ* = 0.419, *p* < 0.001), and a weak, negative correlation with the Well-Being Index (WHO-5) (*ρ* = − 0.177, *p* < 0.001). These findings support the concurrent validity of the Quechua San Martín version of the PHQ-9.

The Quechua San Martín Version of the PHQ-9 demonstrated high internal consistency, with Cronbach’s alpha (α = 0.86) and McDonald’s omega (Ω = 0.87), both exceeding recommended thresholds. The PHQ-9 also achieved acceptable AVE values (0.53), surpassing the theoretical criterion of ≥ 0.50. Additionally, its average factor loadings were adequate (≥ 0.60), indicating that the items adequately represent the underlying construct. Overall, these findings provide strong evidence of reliability and favorable support for the convergent validity of the PHQ-9 (Table 4).

**Table 4:** Reliability and convergent validity indicators for the Quechua San Martín Version of the PHQ-9

	α [IC95%]	Ω (CR) [IC95%]	AVE [IC95%]	Average Loads (± SD)
PHQ-9 (9 items)	0.86 [0.83–0.88]	0.87 [0.84–0.88]	0.53[0.47–0.59]	0.72 ± 0.08

*α* Cronbach’s alpha, *Ω* McDonald’s omega (composite reliability), *AVE* Average Variance Extracted, *SD* Standard Deviation, *95% CI* 95% Confidence Interval

## Discussion

Our results confirm that the PHQ-9 version adapted for San Martín Quechua demonstrates solid psychometric properties, including evidence of validity, reliability, and measurement invariance. Moreover, this represents the first study to adapt a widely used screening instrument to San Martín Quechua, a language spoken in the Peruvian Amazon that has historically lacked culturally and linguistically appropriate psychological assessment tools. This adaptation helps address existing gaps in clinical evaluation resources for populations whose linguistic and cultural contexts have been largely underserved.

Regarding structural validity, the unidimensional model of the Quechua San Martín PHQ-9 mirrors findings from other Quechua psychometric adaptation studies. For instance, a validation study across three Peruvian Quechua varieties (Chanca, Cuzco-Collao, and Central) revealed a similar fit (CFI = 0.990, TLI = 0.987, SRMR = 0.048, RMSEA = 0.071) (17). Similarly, in a bilingual (Quechua–Spanish) sample from Cochabamba, Bolivia, a unidimensional structure was confirmed in both urban and rural settings (CFI = 0.983, TLI = 0.977, RMSEA = 0.069, SRMR = 0.046) [5]. These adaptations include linguistic and cultural adjustments. All Quechua versions, including this one, retained the same factorial structure as the original English PHQ-9 (i.e., unidimensional), based on the criteria for major depression in the Diagnostic and Statistical Manual of Mental Disorders, Fourth Edition (DSM-IV) [39] but its relevance also extends to the DSM-III and DSM-5 [30]. This consistency facilitates standardized use (e.g., using international cut-offs) and enables cross-cultural comparisons.

The evidence for convergent validity is supported by the psychometric performance observed for the PHQ-9. In the present study, the PHQ-9 achieved an acceptable AVE value (0.53), exceeding the recommended threshold of ≥ 0.50, which indicates that the latent construct explains more than half of the variance of its indicators. This finding suggests that the items adequately represent the underlying construct of depressive symptoms. Similar results have been reported in previous validation studies across different cultural contexts. For instance, research conducted among Chinese university students reported AVE values of 0.554 for one factor of the PHQ-9, supporting adequate convergent validity [45]. These findings are consistent with the general criterion that AVE values ≥ 0.50 reflect sufficient shared variance between items and their latent factor, indicating that the construct is well captured by its indicators [22]. Overall, the present results align with prior cross-cultural evidence and support the adequacy of the Quechua San Martín version of the PHQ-9 in capturing depressive symptomatology.

Our findings regarding convergent validity based on relationships with other variables align with those from previous studies. For example, the PHQ-9 demonstrated a strong correlation with the GAD-7 (*r* = 0.68) in a sample of Korean university students [27]. Similarly, a study in Iran found that the PHQ-9 was inversely correlated with the WHO-5 (*r* = − 0.522) and positively correlated with the GAD-7 (*r* = 0.737) [28]. These results highlight the importance of considering comorbidity between depression and anxiety, even in culturally distinct populations, where emotional expression may be shaped by historical, linguistic, and sociocultural factors. The inverse relationship with the WHO-5, for instance, suggests that higher depressive symptoms among Quechua speakers may reflect lower perceptions of well-being, consistent with cross-cultural evidence linking mental health and subjective well-being [20].

Our strong reliability results are like those found in other studies with Indigenous groups, including the adaptation of the PHQ-9 for three types of Peruvian Quechua (Chanca, Cuzco-Collao, and Central) and for Bolivian Quechua [5, 12]. While Cronbach’s alpha can be influenced by item count and sample size, it remains useful as a comparative index [29]. Additionally, omega coefficients based on factor loadings are recommended for more consistent reliability estimation [41].

The Quechua San Martín version of the PHQ-9 demonstrated measurement invariance across groups defined by age, sex, education level, marital status, and type of residence. Our finding is consistent with evidence from other cultural context. The measurement invariance of PHQ-9 has shown measurement invariance in multiethnic European populations, including Dutch, Ghanaian, Turkish, Moroccan, African Surinamese, and South Asian Surinamese groups [19]. In the present study, metric invariance was generally supported across groups, although a marginal deterioration in absolute model fit was observed for age groups, as indicated by a slight increase in RMSEA. Despite this, the overall pattern of results remained acceptable and consistent with invariance assumptions. Importantly, scalar invariance was strongly supported across all grouping variables, with stable CFI values and, in some cases, improvements in RMSEA under more constrained models, suggesting a robust and parsimonious measurement structure. Measurement invariance of the PHQ-9 has also been confirmed in Brazil, where a study of 90,846 participants found invariance across age and sex [16]. In Peru, a study involving 30,449 participants (with an average age of 40.5 years) demonstrated strong invariance across sex, age, education, socioeconomic status, marital status, and urban/rural residence [43]. Likewise, studies in three Peruvian Quechua variants (Chanca, Cuzco-Collao, and Central) [12] and in Bolivian Quechua [5] also confirmed invariance across age, sex, marital status, education level, and area of residence. Taken together, the present results support the presence of at least partial measurement invariance, with particularly strong evidence for scalar invariance. This level of invariance is generally considered sufficient for meaningful comparisons of latent means across groups [35], reinforcing the cross-cultural applicability of the PHQ-9 in Quechua-speaking populations. At the same time, the marginal findings observed for age highlight the importance of cautious interpretation and suggest that future studies may benefit from exploring potential sources of minor non-invariance.

Beyond their statistical adequacy, these findings also have important theoretical implications. From a cross-cultural perspective, invariance should not be interpreted as evidence of complete conceptual equivalence. Rather, it indicates that individuals may respond to items in structurally similar ways, even if the underlying cultural meanings of distress differ [21]. In Indigenous contexts, emotional distress is often embedded in relational, social, and spiritual dimensions, which may not be fully captured by standardized instruments grounded in Western psychiatric frameworks. Therefore, the observed psychometric robustness of the PHQ-9 should be understood as reflecting functional equivalence, rather than full cultural equivalence of the construct [23]. This distinction is important, as it highlights that comparable scores across groups do not necessarily imply identical lived experiences of depression. Integrating these perspectives allows for a more nuanced interpretation of the results and supports the culturally informed use of the instrument in diverse populations.

### Public health implications

Our study marks an important step toward improving mental health services for a historically underserved population. The Quechua version of the PHQ-9 offers a valid and reliable tool that can be implemented in national surveys such as ENDES (Encuesta Demográfica y de Salud Familiar), as well as in community-based mental health programs in the San Martín region, where there is a significant Quechua-speaking population. Its use may facilitate the early detection of depressive symptoms and contribute to more equitable and culturally appropriate care. This initiative aligns with the World Health Organization’s call to ensure equitable access to mental health services for Indigenous and historically excluded populations [47].

### Limitations and strengths

Several strengths and limitations were identified. This is the first cultural adaptation of the PHQ-9 to the San Martín variant of Quechua an extensively used tool to assess depressive symptoms thus including a key Peruvian population often neglected in mental health care efforts. One important limitation of this study is that the PHQ-9 is a written self-report instrument, which presupposes basic literacy skills and may therefore exclude or disadvantage individuals with limited reading proficiency. This constraint is particularly relevant in linguistically and socioeconomically diverse settings, where literacy levels may vary substantially and could introduce selection bias. Future research could address this limitation by incorporating alternative administration modalities, such as interviewer-assisted formats or digital platforms that provide audio delivery of items and capture verbal responses. Emerging technologies, including speech-based interfaces and large language model–supported systems, may offer scalable and culturally adaptable solutions to enhance accessibility while preserving measurement integrity. Furthermore, subsequent studies could assess concurrent validity for a more comprehensive review of the validity.

Furthermore, a limitation concerns the use of the Generalized Anxiety Disorder-7 (GAD-7) and the World Health Organization Well-Being Index (WHO-5), which have not been specifically adapted to the San Martín Quechua context. However, the target population consisted of Quechua-speaking adults with formal education, who were able to read and understand Spanish in addition to their native language. This allowed for the use of validated Peruvian Spanish versions of these instruments. From a psychometric perspective, employing established measures in a linguistically accessible language remains informative for assessing relationships between constructs, particularly when evaluating convergent validity based on associations with related variables. Nonetheless, the absence of culturally adapted versions may introduce potential bias, and future studies should corroborate these findings using instruments specifically validated in the San Martín Quechua context.

## Conclusions

The San Martín Quechua version of the PHQ-9 demonstrated adequate validity and reliability, supporting a well-fitting unidimensional structure and at least partial measurement invariance across sociodemographic groups, with particularly strong evidence for scalar invariance. The instrument also showed convergent validity with anxiety (GAD-7) and well-being (WHO-5) measures, along with adequate internal consistency coefficients.

Overall, the PHQ-9 represents a valuable tool for depression screening among Quechua-speaking populations in San Martín, Peru, and supports meaningful comparisons across groups, contributing to more equitable and interculturally inclusive mental health assessment.

## Data Availability

Doi: 10.5281/zenodo.15676606.
